# Inter-brain plasticity as a biological mechanism of change in psychotherapy: A review and integrative model

**DOI:** 10.3389/fnhum.2022.955238

**Published:** 2022-08-26

**Authors:** Haran Sened, Sigal Zilcha-Mano, Simone Shamay-Tsoory

**Affiliations:** Department of Psychology, University of Haifa, Haifa, Israel

**Keywords:** brain-to-brain coupling, neuropsychology, psychotherapy, synchrony, therapeutic alliance

## Abstract

Recent models of psychopathology and psychotherapy highlight the importance of interpersonal factors. The current review offers a biological perspective on these interpersonal processes by examining inter-brain synchrony—the coupling of brain activity between people interacting with one another. High inter-brain synchrony is associated with better relationships in therapy and in daily life, while deficits in the ability to achieve inter-brain synchrony are associated with a variety of psychological and developmental disorders. The review suggests that therapy improves patients’ ability to achieve such synchrony through inter-brain plasticity—a process by which recurring exposure to high inter-brain synchrony leads to lasting change in a person’s overall ability to synchronize. Therapeutic sessions provide repeated situations with high inter-brain synchrony. This can lead to a long-term increase in the ability to synchronize, first with the therapist, then generalized to other interpersonal relationships, ultimately leading to symptom reduction. The proposed inter-brain plasticity model offers a novel biological framework for understanding relational change in psychotherapy and its links to various forms of psychopathology and provides testable hypotheses for future research. Understanding this mechanism may help improve existing psychotherapy methods and develop new ones.

## Introduction

The effect of the patient-therapist relationship has been the focus of theoretical and clinical writing for the past century. Almost five decades of research suggest that the patient-therapist relationship, as evaluated using self-report measures and behavioral coding systems, is a consistent predictor of treatment outcome (Doran, 2016; Flückiger et al., 2018). In the past decades, researchers have explored more objectively observable indicators of the quality and strength of the relationship. One promising line of research is the study of interpersonal synchrony, defined by Koole et al. (2020) as “the temporal coordination of social agents’ mutual behavioral, physiological, and neurological functions.”

Various approaches have been implemented to evaluate multiple aspects of synchrony between the patient and the therapist during therapy sessions, such as movement energy (Ramseyer and Tschacher, 2011), hormonal (Zilcha-Mano et al., 2021), physiological (Kleinbub et al., 2020), and acoustic markers (Imel et al., 2014). Recently, studies that examined the simultaneous brain activity of patients and psychotherapists have shown that inter-brain synchrony emerges during psychotherapy (Zhang et al., 2018), suggesting that coupling between brain activities of interaction partners may underlie behavioral levels of synchrony and connectedness. Narrative and systematic reviews of the overall literature on synchrony in psychotherapy (Koole and Tschacher, 2016; Koole et al., 2020; Wiltshire et al., 2020; Mende and Schmidt, 2021) have found that a high level of synchrony is associated with the formation of a strong working alliance between the patient and the therapist, as well as with greater treatment efficacy and effectiveness, although there are occasional caveats which call for further research (e.g., Wiltshire et al., 2020; did not find a connection between linguistic synchrony and outcome).

The current review proposes that patient-therapist synchrony might directly increase patients’ ability to establish inter-brain synchrony in the future when interacting with their therapist, and ultimately, with other people.^1^ This can happen through *inter-brain plasticity*; as explained in detail below, inter-brain plasticity (Shamay-Tsoory, 2021) is a phenomenon in which after regions in the brains of two (or more) people are repeatedly activated in close succession (i.e., one immediately after another), connectivity in each brain will become stronger such that these two regions will have a higher chance to be activated together in the future. In synchrony terms, this means that when two people are engaged in an activity involving high inter-brain synchrony, their ability to synchronize will increase, and inter-brain synchrony between them will be greater in the future. We suggest that as psychotherapy is a situation which involves high inter-brain synchrony for extended periods of time, it can trigger inter-brain plasticity.

Importantly, inter-brain synchrony has been associated with better functioning in interpersonal situations and relationships (Hu et al., 2017; Gvirts and Perlmutter, 2020). Thus, improving patients’ ability to synchronize through inter-brain plasticity may be a biological mechanism which can explain how therapy improves patients’ relationships and interpersonal interactions. Many forms of psychopathology are associated with interpersonal difficulties (Girard et al., 2017) and multiple theoretical frameworks of psychopathology and psychotherapy revolve around interpersonal relationships. Examples include contemporary integrative interpersonal theory (CIIT) (Hopwood et al., 2021), relational and intersubjective psychoanalytical theory (Mitchell and Aron, 1999; Stolorow et al., 2014), and interpersonal psychotherapy (Blagys and Hilsenroth, 2000), among others; even when they are not the focal point of treatment, the role of interpersonal components is often recognized, such as in recent research on CBT (Castonguay et al., 2018; Kazantzis et al., 2018). Inter-brain plasticity may help explain some of these key interpersonal processes on a biological level.

We begin by briefly detailing our method and discussing the definitions of synchrony. We then introduce inter-brain synchrony and review studies linking it with prosocial behavior, deeper interpersonal relationships, and stronger therapeutic alliances. We continue by describing inter-brain plasticity and review studies documenting its occurrence. We then review clinical literature showing how various psychological and neurological disorders are associated with low inter-brain and behavioral synchrony, and how, following therapy the synchronization ability may increase. We conclude by presenting a model for inter-brain plasticity in psychotherapy. We discuss implications for clinical research and practice, as well as addressing alternative explanations for our findings and providing directions for future research.

## Methods

The current review is a non-systematic narrative review. This approach was chosen as our aim is to demonstrate how indirect evidence from a variety of research programs possibly points to a phenomenon. Such broad discussion of an evolving concept, as opposed to a review of literature on an established topic, is better suited to a narrative review (Collins and Fauser, 2005). In a more practical sense, as we integrate findings from multiple lines of research, performing a systematic review of each one of them would be infeasible.

Still, following Ferrari’s (2015) suggestion to include some methods of systematic review in narrative reviews, we detail some attempts we made to stratify our article search methodology. In general, literature searches were performed on Google Scholar and PsycArticles. Each search was repeated once using brain-specific terms (“Inter-Brain Synchrony” OR “Inter-Brain Synchronization” OR “Brain Coupling”^2^) coupled with a relevant additional term (e.g., “Psychotherapy,” “Depression”), and once simply using “Synchrony” to examine behavioral and other forms of synchrony. When discussing plasticity, we included either studies which contrasted synchrony at multiple timepoints, or who correlated synchrony with an individual difference variable which could indicate differences in repeated exposure to a situation, e.g., experienced vs. novice professionals (Zhang et al., 2020), people with existing relationships vs. strangers (Kinreich et al., 2017), different types of repeated contact with caregivers during development (Yaniv et al., 2021). However, the large number of searches required to cover all of the topics discussed meant that we could not systematically categorize all results of each search. To somewhat counteract possible biases, we highlight existing systematic reviews and meta-analyses on specific topics whenever possible.

## Interpersonal synchrony: Definitions

As mentioned above, the definitions of synchrony (Koole et al., 2020), imply that the phenomenon in question must have some temporal variance, which is shared between participants; the specific behavior, physiological or neural measure at a specific point in time does not have to match. For example, two people standing on a basketball court would not be considered synchronized in movement just for performing the same action, as there is no variance in behavior over time. However, if they started throwing the ball back and forth, they would be considered synchronized in movement; although they are never simultaneously performing the same action, their actions are perfectly correlated over time (whenever person A is throwing, person B is catching, and vice versa).

From a temporal perspective, there are multiple subtypes of synchrony with different definitions of “temporal coordination” (For a full review, see Butler, 2011). One important distinction is between trend, concurrent and lagged synchrony (Helm et al., 2018). Trend synchrony is a correlated trend between people in a measure (behavioral, physiological or neural) over a long period of time. Concurrent synchrony is a common fluctuation of the measure around a trend. Lagged synchrony is similar to concurrent synchrony, but with one of the participants “leading” the other, i.e., one participants’ measures are correlated with the other participants’ measures at a previous time-point. Studies of interpersonal synchrony in conversation settings, as the ones detailed below, generally measure concurrent synchrony, while allowing for short lags in either direction (e.g., by averaging results with lags between –5 and +5 s; Paulick et al., 2018). Short lags must be accounted for as they may stem from a variety of reasons, including small discrepancies in measurement timing, differences in inherent delays such as an approximately 6 s delay between neuronal activity and blood response (Liao et al., 2002), and differences in reaction times and in movement speeds between participants. Trend synchrony is of less theoretical interest—as detailed above, theories of synchrony in interpersonal interaction focus on the moment-to-moment interaction between people, and not on general similarity over long periods of time. Another common distinction is between in-phase synchrony, in which participants’ levels of measures are positively correlated (e.g., dancers performing the same moves at the same time), and anti-phase synchrony, in which participants’ actions are negatively correlated (e.g., a conversation in which whenever one person talks the other is silent). As interpersonal interaction studies must account for lags in either direction, the distinction between in-phase and anti-phase synchrony is murkier, and they are usually aggregated.

How do people establish synchrony with one another? Prominent theories highlight the importance of being able to perceive each other’s behavior, and by having a consistent reaction which is perceived by the other person (Hasson et al., 2012; Wheatley et al., 2012). Thus, the occurrence of synchrony is an indicator of participants’ ability to perceive each other, and their willingness and ability to react to each other. Once synchrony has been established, it also has the direct benefit of making predictions of the other person easier, freeing cognitive resources for other tasks (Hoehl et al., 2021). Indeed, multiple systematic reviews and meta-analyses (Rennung and Göritz, 2016; Mogan et al., 2017; Czeszumski et al., 2022) have linked behavioral and neural synchrony to a variety of positive outcomes.

Importantly, while we could find no studies linking inter-brain synchrony and negative relational outcomes, there are studies from other modalities which show negative effect. Systematic reviews and meta analyses of physiological synchrony in the autonomous nervous system show mixed results (Palumbo et al., 2017; Mayo et al., 2021). In psychotherapy, while reviews find general positive effects (Koole et al., 2020; Wiltshire et al., 2020), some studies of behavioral synchrony have reported mixed results (Ramseyer, 2020; Tschacher and Meier, 2020). As mentioned above, a study by Paulick et al. (2018) has even found negative associations between behavioral synchrony and outcome for patients with anxiety disorders. A prominent explanation behind these more mixed results is context dependence (Danyluck and Page-Gould, 2019). Indeed, it could be the case that in some cases, a flexible balance between synchrony and non-synchrony is more important than constant high synchrony (Mayo and Gordon, 2020).

While these mixed results should be taken in mind, the current review follows the aforementioned systematic reviews and meta-analyses which show overall positive outcomes of interpersonal synchrony. Still, we certainly do not expect increased inter-brain synchrony to be a panacea, and we expect that as research on inter-brain plasticity progresses specific disorders, subtypes of synchrony, or session contexts may emerge as contra-indications.

## Inter-brain synchrony, relationships, and therapeutic alliance

Inter-brain synchrony, also referred to as brain-to-brain coupling, represents synchronized activity patterns between the brains of two (or more) people. Inter-brain synchrony is a widely observed phenomenon, thought to occur through the transfer of various signals between brains using external channels such as speech, gestures, and facial emotions (Hasson et al., 2012). It is usually examined using hyperscanning—the simultaneous acquisition of the cerebral data from two subjects (Montague et al., 2002; Babiloni and Astolfi, 2014). Current hyperscanning methods include functional near-infrared spectroscopy (fNIRS) (Ferrari and Quaresima, 2012), dual-EEG (Liu et al., 2018), and more rarely, fMRI (Misaki et al., 2021).

Inter-brain synchrony is associated with several positive interpersonal outcomes. In one study in which participants played a prisoner’s dilemma game, participants who displayed greater inter-brain synchrony (as measured via dual-EEG) were more cooperative (Hu et al., 2017). In a study of teams engaged in cooperative problem solving, inter-brain synchrony measured using EEG hyperscanning predicted cooperative behavior even beyond self-reported team identification (Reinero et al., 2020). Gvirts and Perlmutter (2020) propose a mechanism for this effect, suggesting that inter-brain synchrony may help increase mutual attention and social alignment—the tendency of individuals to align their motions, emotions and cognitions (Shamay-Tsoory et al., 2019). Recent studies have examined these questions using dyadic neurofeedback paradigms, in which participants’ brain activity is visualized (e.g., by displaying coherence metrics between two people’s EEG readings; Chen et al., 2021), allowing them to see whether it is synchronized or not. Participants who are instructed to use this feedback to increase their inter-brain synchrony over time are able to do so (e.g., Susnoschi Luca et al., 2021). Dyadic neurofeedback studies in humans (Müller et al., 2021) and in pigeons (Yang et al., 2020) have demonstrated causal links between inter-brain synchrony and prosociality; the researchers increased inter-brain synchrony using dyadic neurofeedback and demonstrated that this increased synchrony was associated with more pro-social experiences (Müller et al., 2021) and behavior (Yang et al., 2020).

**FIGURE 1:**
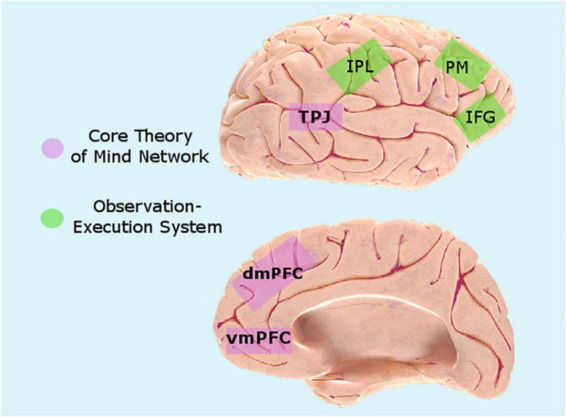
Target regions for inter-brain plasticity. Figure based on brain images © Society for Neuroscience, copied with permission from www.brainfacts.org.

Research on inter-brain synchrony shows that it may occur between various brain networks. The literature points to two networks which may be especially relevant: the theory of mind network and the observation-execution system (see Figure 1).

The theory of mind network involves reasoning about, considering and simulating the mental states of others and is key to social interaction (Rilling et al., 2004). Theory of mind is a broad term, encompassing various processes in many brain regions. A “core” theory of mind network (Carrington and Bailey, 2009; Schurz et al., 2014) encompasses the temporal-parietal junction (TPJ) and the medial prefrontal cortex [both the dorsomedial (dmPFC) and the ventromedial (vmPFC) prefrontal cortex, with some differentiation between tasks; Shamay-Tsoory and Aharon-Peretz, 2007]. Both regions have been shown to be activated in a wide range of theory of mind related tasks (Tamir and Mitchell, 2010; Schurz et al., 2017; Paracampo et al., 2018), and comprise part of the cognitive empathy system (Abu-Akel and Shamay-Tsoory, 2011).

Another system that may support emotional communication is the observation-execution system. This system was identified in the inferior frontal gyrus (IFG) and the inferior parietal lobule (IPL) and premotor (PM) cortices, with the IFG pertaining to motor representations of actions, whereas the IPL is linked to the actual sensory-to-motor mapping of visual input, and own-body vs. other coordinates. This system is activated in multiple interpersonal contexts, such as emotional contagion, vicarious pain, and emotion observation (Shamay-Tsoory et al., 2019), and overlaps with the emotional empathy system (Abu-Akel and Shamay-Tsoory, 2011).

Synchrony in both systems has been associated with improved communication and cooperation and with better relationships. A recent meta-analysis found evidence of synchrony in the temporo-parietal and prefrontal cortex during collaborative tasks (Czeszumski et al., 2022). More specifically, synchrony in the TPJ and the medial prefrontal cortex was found during collaborative tasks such as drawing (Xie et al., 2020) and problem solving (Lu et al., 2019), and in freeform conversations between romantic couples, as opposed to conversations between strangers (Kinreich et al., 2017).

As for the observation-execution system, an increase in inter-brain synchrony of the left IFG, compared to rest, was found during coordinated face-to-face dialog between partners (Jiang et al., 2012). Dual-EEG studies further confirm the relevance of inter-brain synchrony in the alpha/mu band (8–12 or 13 Hz) that is considered a biomarker of the observation-execution system (Astolfi et al., 2010; De Vico Fallani et al., 2010), during imitation (Dumas et al., 2010). Such synchrony also predicts the level of analgesia during handholding (Goldstein et al., 2018).

Finally, a small number of studies were able to demonstrate links between inter-brain synchrony and psychotherapy, and that synchrony was associated with high levels of working alliance. Zhang et al. (2018) used fNIRS to perform brain imaging on therapist-patient dyads in a single session. Thirty-four students who presented to a college counseling center (with no specific diagnosis) were randomly assigned to a single therapy session or to a social chatting session. Therapists provided therapy in an integrative orientation (Stricker and Gold, 2008). Inter-brain synchrony in the right temporo-parietal junction (rTPJ) was higher in the therapy condition. These findings indicate that inter-brain synchrony is higher in treatment sessions than in day-to-day social encounters. Importantly, within the therapy condition, inter-brain synchrony and working alliance were associated—higher inter-brain synchrony was recorded for participants who reported a stronger working alliance. In an additional study by the same team, Zhang et al. (2020) found that experienced, licensed therapists developed significantly stronger inter-brain synchrony with their patients than novice therapists (First-year graduate students with 15–24 h of experience), as well as a stronger working alliance reported by the patient. For experienced therapists, but not for novice ones, inter-brain synchrony was associated with a stronger working alliance. This indicates that therapists’ training may improve their ability to create strong inter-brain synchrony in a session. Lecchi et al. (2019) examined 14 therapist-patient dyads in single sessions using dual-EEG. Patients reported low mood or anxiety issues during the preceding fortnight. High interbrain synchrony was associated with greater congruence between patient and therapist ratings of the working alliance, and with high patient working alliance ratings. Interestingly, synchrony was the same whether sessions were conducted in person or through video conference. Thus, in both studies inter-brain synchrony was associated with better therapeutic relationships in single sessions.

Importantly, indirect insight into the causes and effects of inter-brain synchrony can also be gained from the broader behavioral and physiological synchrony literature. While behavioral synchrony and neural synchrony are not identical, they have been shown to coincide (Dumas et al., 2010), with neural synchrony having a causal influence on behavioral synchrony (Novembre et al., 2017). Thus, behavioral synchrony may be seen as an (imperfect) proxy measure for inter-brain synchrony, and in areas of research where studies explicitly measuring inter-brain synchrony are scarce we discuss behavioral synchrony studies as well.

## Inter-brain plasticity

As detailed above, inter-brain synchrony is associated with prosocial behavior and better relationships, within psychotherapy and without. This raises the question—can a person’s general ability to achieve inter-brain synchrony be changed? At the neural level, existing research has established that connectivity between brain regions in a single brain can change. Experience-dependent short- and long-term changes in connectivity in several networks (i.e., changes in inter-*system* synchrony) have been reported to underlie various types of learning (e.g., Garrido et al., 2009). According to the spike-timing-dependent plasticity (STDP) principle, which has been widely supported (Caporale and Dan, 2008), when two neurons, or whole brain regions, fire one after another in close succession, synaptic strength will increase. For example, a study of infants aged 5–8 months (King et al., 2021) has shown that exposure to language was associated with higher connectivity between regions in the auditory cortex, the left inferior frontal gyrus (IFG), and the bilateral superior temporal gyrus (STG). Recent studies have managed to purposefully activate such plasticity by using transcranial magnetic stimulation (TMS) to stimulate two brain regions in rapid succession (Suppa et al., 2022); for example, in one study researchers were able to improve participants’ hand dexterity after stroke by stimulating the cerebellum and the motor cortex (Rosso et al., 2022).

While there are many cellular-level pathways which can lead to STDP, one of the most studied ones is through N-methyl-D-aspartate receptors (NMDAR), which can only be activated by the pre-synaptic neuron when the post-synaptic neuron is depolarized—allowing it to detect the specific timing of activation typical to STDP learning. When activated, the NMDAR releases large amounts of calcium, which in turn causes long-term potentiation of the synapse (Malenka and Bear, 2004; Caporale and Dan, 2008). Interestingly, this process may be modulated by various neurotransmitters, including Oxytocin (Lin and Hsu, 2018) a neurohormone associated with the regulation of social interactions (Froemke and Young, 2021).

The notion of STDP was recently expanded by taking an inter-brain approach to plasticity (Shamay-Tsoory, 2021). The inter-brain (or second brain) approach (Redcay and Schilbach, 2019) views multiple brains of interacting individuals as parts of an extended network in which nodes, or units, represent different individuals (Hari and Kujala, 2009). Thus, the concept of inter-brain plasticity posits that in a manner similar to regions in the same brain, when regions in two brains are activated in close succession, as is the case in inter-brain synchrony, synchrony between them will grow stronger.

**FIGURE 2:**
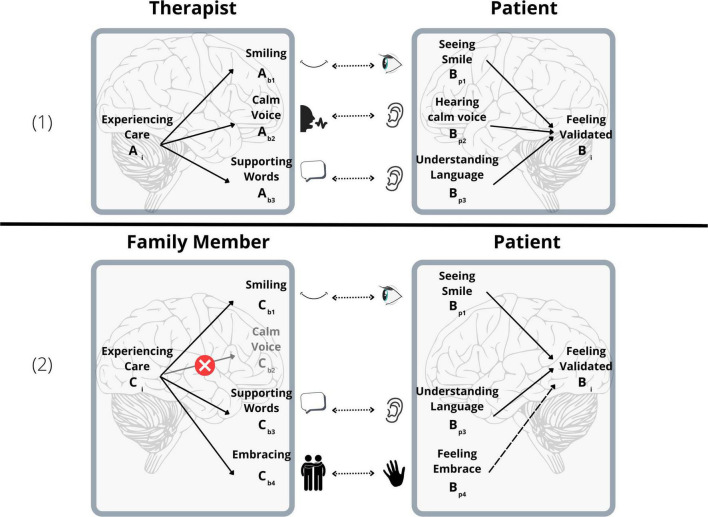
(1) Inter-brain pathways, including neuronal and behavioral links. As neuronal pathways grow stronger, if the behavioral links stay constant, inter-brain pathways grow stronger as a whole. The link between two mental states involves multiple inter-brain pathways, which might all grow gradually stronger through inter-brain plasticity. (2) After a period of therapy, in a new encounter with a family member, not all pathways are present (e.g., the family member uses an excited, no calm, voice). However, enough pathways are present to link the two mental states. This can allow a new pathway (e.g., through embracing), which was not present during therapy, to start forming.

Importantly, inter-brain plasticity as a concept does not posit a new biological or physical fact beyond single brain plasticity, and the possibility of interpersonal communication. Consider person A’s inner mental state (A_*i*_) leading, through neural processes in their own brain, to specific behavior (A_*b*_). For example, as depicted in panel 1 of Figure 2, a therapist might experience empathy and caring toward a patient, leading them to smile. That behavior is then perceived by person B (e.g., through vision), and registered in their own brain (B_*p*_)—the patient sees the therapist’s smile. Through their own neural processes, that may lead to changes in their own inner mental state (B_*i*_)—seeing the therapist smile leads the patient to feel validated. As long as this process is repeated, we would expect the connections A_*i*_-A_*b*_ and B_*p*_-B_*i*_ to grow stronger, through plasticity processes within a single brain. Assuming that person B’s perceptual capacities (i.e., the connection A_*b*_-B_*p*_) have stayed the same over repeated interactions, this would naturally lead the direct inter-brain association A_*i*–_B_*i*_ to increase (via the pathway A_*i*_-A_*b*_-B_*p*_-B_*i*_). Of course, in actual interpersonal relationships, person A’s inner mental state might be reflected in a variety of behaviors. For example, as depicted in panel 1 of Figure 2, a therapist caring for her patient may smile, support them verbally, or adopt a relaxed speaking tone. The notion of inter-brain plasticity allows us to avoid cataloging changes in numerous behavioral-perceptual pathways (A_*i*_-A_*b*1_-B_*p*1_-B_*i*_, A_*i*_-A_*b*2_-B_*p*2_-B_*i*_, etc.), focusing instead on the gradually increasing association between a single pair of mental states (A_*i*_-B_*i*_). Note that this example does not involve or require behavioral synchrony (e.g., both people smile)—only the possibility of perception (e.g., when one person smiles, the other person is able to see their smile). Of course, sometimes synchronized mental states might lead to synchronized behavior (e.g., synchrony between a therapist feeling empathy and a patient feeling validated might lead them both to smile).

Several lines of research have demonstrated inter-brain plasticity in various types of interpersonal interaction. Yaniv et al. (2021) have shown in a longitudinal study that the ability to *behaviorally* synchronize increases throughout development, from infancy to young adulthood. Babies who were carried to full term had a better ability to synchronize at all ages. Importantly, babies born pre-term whose mothers employed kangaroo care (increased amounts of skin-to-skin touch between mother and baby) at infancy had a higher capability to synchronize throughout development than matched controls whose mothers did not employ this method. This shows that care-taking behaviors can have long-term effects on the capability to synchronize, at least behaviorally, suggesting that inter-brain plasticity may have taken place.

With respect to relationships, research has shown that inter-brain synchrony is stronger in closer relationships. Multiple studies have shown that inter-brain synchrony is correlated with social closeness (Dikker et al., 2021); that romantic partners have higher inter-brain synchrony with each other than strangers (Kinreich et al., 2017), and that students who feel closer to their teachers are also more synchronized with them (Bevilacqua et al., 2019). These findings support the notion that inter-brain plasticity occurs over the course of the relationship, gradually increasing inter-brain synchrony.

Studies of more specific interpersonal interactions also support this idea. For example, changes in brain synchrony have been documented after a teaching session (Zheng et al., 2020), and therapy was shown to cause changes in behavioral synchrony (e.g., Venuti et al., 2017; Galbusera et al., 2018). Thus, series of professional encounters with a teacher or a therapist can change people’s ability to synchronize. Importantly, there are indications that this kind of improvement can generalize to interactions with other people. In addition to the aforementioned study showing that experienced therapists have stronger inter-brain synchrony than novice ones (Zhang et al., 2020), a study of teaching sessions has shown that expert teachers synchronize better with new students than novice teachers (Sun et al., 2020). These two studies suggest that as teachers and therapists gain experience, inter-brain plasticity occurs.

A major consideration regarding inter-brain plasticity is that in order to lead to significant change in patients’ lives, it must involve consolidation and generalization. Consolidation is the process through which new memories, which are initially susceptible to be overwritten with new information, become stable for long periods of time (McGaugh, 2000). For inter-brain plasticity in therapy, this would mean that increases in synchrony achieved in one session would be retained in future sessions.

Generalization (Ghirlanda and Enquist, 2003) is the process through which the response to one set of stimuli becomes associated with a new set of similar stimuli. Synchrony is highly context dependent (see above for examples concerning synchrony in cooperative vs. non-cooperative situations, as well as in therapy vs. in small talk). Thus, when discussing changes in a person’s “ability to synchronize,” we are referring to changes in the amount of synchronization they tend to achieve in a specific set of contexts. We expect changes due to inter-brain plasticity to be limited at first to the exact context in which the initial synchronous experiences occurred. However, this could gradually generalize to similar situations.

For inter-brain plasticity in therapy, this would mean that changes in patients’ ability to achieve inter-brain synchrony *in therapy with their specific therapist* would lead to changes in their ability to synchronize (a) *with people other than their therapist* and (b) *in different contexts*, such as various day-to-day interactions. Extending the earlier example, generalization may take the following form: Following a variety of interactions with a therapist A (A_i_-A_b1_-B_p1_-B_i_, A_i_-A_b2_-B_p2_-B_i_, …), patient B meets another person, C, in a different context, such as a social meeting with a family member, as detailed in panel 2 of Figure 2. Although C may have a mental state analogous to one encountered in therapy (C_i_), as this is a different person in a different context, C might only engage in a subset of the behaviors experienced in the interaction with A (C_i_-C_b1_-B_p1_-B_i_, C_i_-C_b3_-B_p3_-B_i_, but not C_i_-C_b2_-B_p2_-B_i_). For example, while both the therapist and the family member might smile at the patient and support them verbally when they experience caring for them, the therapist might have been speaking in a calm and reassuring tone of voice, which the family member does not use. However, as this subset of associations (B_p1_-B_i_,B_p3_-B_i_, …) were strengthened for B in therapy, the association C_i_-B_i_ will still be stronger than it might have been before therapy. What if C’s mental state is also reflected in an entirely new behavior (e.g., C_i_-C_b4_-B_p4_), which may have been absent from therapy, such as embracing the patient? Considering that B’s internal state representation B_i_ is already activated through the pathways which were trained in therapy, B_p4_ and B_i_ will be activated at the same time. According to the STDP principle, we expect that this will lead the pathway B_p4_-B_i_ to become stronger, and ultimately B will be able to synchronize with C through this new behavior, which was not present in therapy at all.

While a full review of consolidation and generalization is beyond the scope of this article, one of the major findings of the literature concerning consolidation and generalization is the spacing effect—the fact that consolidation and generalization are stronger when information is presented repeatedly in spaced intervals (Smith and Scarf, 2017). As psychotherapy is often delivered in intervals (e.g., weekly sessions), it has a high potential to encourage consolidation and generalization.

## Behavioral and inter-brain synchrony in psychopathology

Many forms of psychopathology are associated with a reduced ability to achieve inter-brain synchronization in various contexts. Therapy can help patients mitigate this deficit. The following section reviews the transdiagnostical role of deficiencies in patients’ ability to synchronize, and evidence that psychotherapy improves this ability.

Autistic spectrum disorder (ASD) has often been associated with reduced interpersonal synchrony, including reduced inter-brain synchrony (McNaughton and Redcay, 2020). Autistic individuals repeatedly exhibit difficulties in tasks that involve movement synchrony (Feldman, 2007; Fournier et al., 2010; Marsh et al., 2013; Fitzpatrick et al., 2016; Cheng et al., 2017). Concerning neural synchrony, two hyperscanning fMRI studies reported that autistic individuals show reduced brain-to-brain coupling of the IFG compared to typically developing (TD) individuals (Tanabe et al., 2012; Wang et al., 2020). A recent fNIRS hyperscanning study found similarly reduced synchrony in the TPJ during a conversation for autistic individuals as compared to TD individuals (Quiñones-Camacho et al., 2021).

Importantly, some studies have shown that various forms of therapy can improve the ability of autistic individuals to synchronize. For example, autistic children treated with dog-assisted therapy (Griffioen et al., 2020) showed more synchrony with the therapy dog’s movements. In a study of music therapy for autistic children, not only did interpersonal synchrony of emotion and behavior improve over the course of therapy, but this improvement generalized to synchrony with an unknown adult administering a diagnostic interview (Venuti et al., 2017). This suggests both inter-brain plasticity and generalization following therapy.

Borderline personality disorder (BPD) has also been associated with reduced synchrony. Individuals with BPD showed reduced behavioral synchrony during a music improvisation task (Foubert et al., 2017). A neuroimaging study revealed reduced inter-brain synchrony in the rTPJ in conversations between individuals with BPD and healthy controls as opposed to conversations between two healthy controls (Bilek et al., 2017). Crucially, the study found that individuals with BPD *in remission* had the same synchrony capability as healthy controls, suggesting that inter-brain plasticity has occurred.

Symptoms of schizophrenia have been associated with reduced movement synchrony (Kupper et al., 2015) and overall interpersonal behavioral coordination (Dean et al., 2021). Interestingly, in a study of human-robot interactions, positive social feedback helped healthy controls, but not schizophrenia patients, improve their motion synchrony with a robot, indicating that it might be especially difficult to induce inter-brain plasticity in such patients (Cohen et al., 2017). Nevertheless, in accordance with inter-brain plasticity as a mechanism of change, a study of body-oriented psychotherapy found that the ability of patients with schizophrenia to achieve movement synchrony increased after therapy (Galbusera et al., 2018).

Major depressive disorder (MDD) has been associated with synchrony deficits, such as reduced movement and facial synchrony in clinical interviews (Altmann et al., 2021). Mothers with a history of major depression were shown to be less synchronized with their children both behaviorally (Granat et al., 2017) and physiologically (Woody et al., 2016). In the context of psychotherapy, a recent study has shown that coupling of the levels of the neurohormone oxytocin between the patient and the psychotherapist is associated with better psychotherapy outcomes for depression (Zilcha-Mano et al., 2021). Another recent study has demonstrated that patients diagnosed with a depressive disorder were less synchronized behaviorally with their therapists than patients with anxiety disorders (Paulick et al., 2018). As with other conditions, the latter study demonstrated that for depressed patients, behavioral synchrony increased as treatment progressed, suggesting that inter-brain plasticity may have occurred.

Social anxiety has also been linked to reduced movement and heart rate synchrony (Asher et al., 2020, 2021). In a recent study, higher movement synchrony was associated with better treatment outcomes for clients with social anxiety (Altmann et al., 2020). However, studies examining anxiety disorders in general, without looking at specific disorders, reported different results; mothers with anxiety disorders had increased synchrony with their children (Granat et al., 2017) and patients with anxiety disorders demonstrated *reduced* synchrony following cognitive-behavioral psychotherapy (Paulick et al., 2018). As the evidence for the significance of behavioral synchrony in anxiety disorders is mixed, special care is required when examining the role of inter-brain plasticity in psychotherapy for these disorders.

## The inter-brain plasticity in psychotherapy model and its implications

**FIGURE 3:**
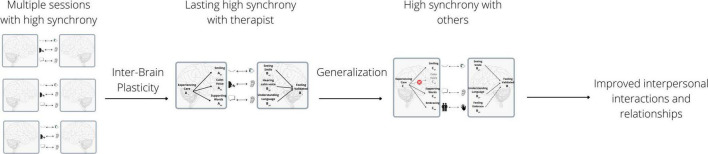
The inter-brain plasticity in psychotherapy model. Multiple sessions with high therapist-patient synchrony improve synchrony with therapist. This generalizes to improved synchrony with others, which allows for improved interpersonal interactions and relationships.

To integrate the various lines of research reviewed above, we propose a model of inter-brain plasticity in psychotherapy, detailed in Figure 3. We posit that (1) Psychotherapy involves high inter-brain synchrony between patients and therapists; (2) by helping patients repeatedly achieve high inter-brain synchrony, therapy increases patients’ ability to synchronize with the therapist, and ultimately with others, through inter-brain plasticity and generalization; and (3) this increase in the ability to synchronize underlies some of the beneficial effects of psychotherapy.

As evidence for claim (1), we have reviewed both direct studies (Zhang et al., 2018; Lecchi et al., 2019) showing high inter-brain synchrony during psychotherapy, as well as general research linking inter-brain synchrony with strong relationships. As evidence for claim (2), we have reviewed studies showing increases in inter-brain and behavioral synchrony over the course of parent-child and peer relationships; over the course of learning in a classroom; and over the course of psychotherapy, as evident in studies showing higher behavioral synchrony after, as opposed to before, psychotherapy. As evidence for claim (3), we have reviewed studies showing that symptom levels of various disorders are correlated with inter-brain and behavioral synchrony deficits, and that changes in behavioral synchrony over the course of therapy are correlated with changes in symptoms (Paulick et al., 2018).

While the model focuses on therapeutic relationships, it does not preclude inter-brain plasticity from happening outside of therapy—to the contrary, we have reviewed many studies showing inter-brain plasticity in other contexts; the proposed model simply focuses on how inter-brain plasticity might operate in a therapeutic setting. However, it does suggest that therapy can potentially lead patients to more inter-brain plasticity than other activities, for two main reasons. First, it posits that therapy is a high-synchrony activity (claim 1). Second, having a positive interpersonal interaction for about an hour with no distractions can be rare in many people’s lives; people with synchrony deficits, which are common in many disorders (claim 3), may find it especially difficult to establish relationships in which they have such long, positive, high-synchrony interactions with others regularly. A therapeutic setting allows them to have this type of interaction week after week.

### Implications

The inter-brain plasticity in psychotherapy model has three major implications for psychotherapy research and practice. First, as inter-brain plasticity stems out of single brain plasticity, it follows that biological conditions which affect plasticity will have corresponding influence on the efficacy of psychotherapy. Some of these conditions may be difficult or impossible to alter—for example, old age, as well as some neurological conditions, are associated with reduced plasticity (Pascual-Leone et al., 2011). However, some conditions may be alterable, and could be incorporated alongside psychotherapy to increase its effectiveness. For example, having enough sleep (Abel et al., 2013) and engaging in physical activity (Erickson et al., 2012) may increase inter-brain plasticity.

A second implication of the model is that it suggests that directly inducing inter-brain synchrony may have beneficial, long-lasting effects on patients’ interpersonal relationships and interactions through inter-brain plasticity. Several methods have been demonstrated to increase inter-brain synchrony. For example, listening to music together was shown to increase inter-brain synchrony (Abrams et al., 2013; Khalil et al., 2022). Performing synchronized arm movements was shown to improve synchrony in a later teaching session, demonstrating that synchronizing can precede the interpersonal interaction (Nozawa et al., 2019). In another study, inter-brain synchrony was increased by administering Oxytocin (Mu et al., 2016). Other researchers examine the capabilities of dyadic neurofeedback to increase interpersonal synchrony and influence interpersonal interactions (Duan et al., 2013; Kovacevic et al., 2015; Dikker et al., 2021; Müller et al., 2021). Finally, in a recent study (Pan et al., 2020b) researchers used dual transcranial alternate current stimulation (tACS) to manipulate synchrony between music instructors and students. Increasing participants’ inter-brain synchrony improved learning compared to controls. Interestingly, this improvement was mediated by increased interpersonal behavioral synchrony. Similar manipulations should be examined in the context of psychotherapy—either by incorporating synchrony increasing exercises such as joint music listening into psychotherapy sessions, or by complimenting psychotherapy with separate sessions incorporating dyadic neurofeedback or synchronized movement. While existing models of inter-brain synchrony may also provide mechanisms through which increasing synchrony in-session could improve outcomes (e.g., by improving the therapeutic alliance; Zhang et al., 2018), the inter-brain plasticity model suggests an additional mechanism which may underlie this phenomenon; it also uniquely predicts that separate synchrony-inducing sessions with others, even if they do not include therapy, would be beneficial, as they would also increase patients’ ability to synchronize and ultimately lead to better interpersonal interactions and relationships.

A final implication is that inter-brain plasticity could serve as a measure of therapy improvement. While for clinical purposes the high cost of imaging devices may render them impractical, in research settings measuring inter-brain synchrony and plasticity can serve as a measure which is less affected by subjective biases than self-report; imaging during psychotherapy sessions has the additional advantage of providing a continuous measure with which the effects of specific moments in the session may be examined.

## Alternative explanations and caveats

### Behavioral mechanisms of change

The proposed model does not replace behavioral models of change, such as mediations of therapeutic change by the working alliance (Baier et al., 2020). In fact, for inter-brain plasticity to occur, it must be reflected behaviorally, as behavior (and the perception of it by the other person) is the only way for information to be conveyed between two brains. However, as detailed above, this biological perspective can help understand the contribution of biological factors to therapeutic change (e.g., sleep and physical activity), design supplementary biological interventions (e.g., inducing synchrony by listening to music), and incorporate biological measures into psychotherapy research.

### Inter-brain plasticity as a confound

While any psychological change must be reflected somehow in the brain, one could argue that changes occurring during psychotherapy are better understood through a single-brain perspective, and that changes in inter-brain synchrony are mere confounds. Indeed, previous neuroscientific research on change in psychotherapy has identified changes in patients’ brains over the course of therapy (Barsaglini et al., 2014). We agree that some of the effects of psychotherapy would be better construed as single brain plasticity. For example, a recent study has identified changes in the neural reaction to spiders after exposure therapy (Rosenbaum et al., 2020). However, we believe that when attempting to document the relational effect of psychotherapy from a neural perspective, a single brain approach would require documenting neural reactions to an extremely wide range of relational stimuli (words, gestures, facial expressions, body postures, etc.). Recognizing that this range of stimuli stem from the presence of another person (and another brain) is a much more parsimonious and allows for a more informative explanation. A recent study supporting this notion attempted to compare single and dual brain explanations in a teaching paradigm (Pan et al., 2020a). Dual brain information was significantly better than single brain information in identifying the teaching style employed in a study session.

Another alternative explanation could be that inter-brain plasticity is a measurement confound, e.g., that it simply reflects statistical properties of measurement, or effects of double measurement of neural data. However, there is evidence that this is not the case. First, some studies show that people who have underwent interpersonal processes which should, according to the model, result in inter-brain plasticity, demonstrate increased synchrony when measured in a single measurement. As detailed above, experienced teachers (Sun et al., 2020) and therapists (Zhang et al., 2020) achieved stronger synchrony than their novice counterparts; in the context of specific relationships, people in stronger relationships exhibit more synchrony (Kinreich et al., 2017; Dikker et al., 2021); and participants with borderline personality disorder in remission show higher synchrony that participants with an active disorder (Bilek et al., 2017). Second, studies looking at synchrony over short time frames (e.g., within a single long interaction) often show that synchrony is stable or even declines (Reinero et al., 2020; e.g., Galbusera et al., 2018). If increases in synchrony were purely due to measurement, we would expect synchrony to increase over short time-frames, perhaps even more than after long periods of time with no measurement (as is the case with evidence of inter-brain synchrony). Still, to fully reject these alternative explanations, future studies should be performed in which the number of measurements varies between participants, to demonstrate that it is not driving inter-brain plasticity.

## Future directions

### Full model tests

While we have reviewed evidence for the various claims made by the proposed model, no study has directly tested the complete model. Future studies should measure inter-brain synchrony over the course of psychotherapy, ideally both between patients and therapists and between patients and others (to establish generalization). We expect inter-brain synchrony to increase over the course of therapy, and to be associated with symptom reduction. We expect these increases to be associated with the quantity of synchronous experiences (i.e., number of sessions). We also expect such increases to be associated with the levels of synchrony in each session, such that high synchrony in a session would lead to higher gains in synchrony. However, researchers should take care to avoid ceiling effects, as people with a high ability to synchronize might not have much room to improve. Finally, integrating external methods to improve synchrony (e.g., having patients and therapists listen to music together before sessions) could help demonstrate causality.

### Moderating factors

As cited above, reduced ability to synchronize is a feature of multiple psychological conditions. However, these conditions might respond differently to improvement in synchrony ability via inter-brain plasticity. In some conditions, difficulties in synchrony may be core features, underlying the condition; in those conditions, improving the ability to synchronize can lead to general psychological change. In other conditions, difficulties in synchrony may be the result of other processes; in these conditions, while improving the ability to synchronize may carry some benefits, these may be rather limited. Importantly, some conditions, such as anxiety disorders, might be characterized by over-synchrony (Paulick et al., 2018) although, as detailed above, evidence is inconclusive. If this is indeed the case, methods to *avoid* increases in synchrony, or to better adapt the level of synchrony to the specific patient, should be developed.

Another important possible moderator is the type of treatment—both the general treatment modality (e.g., psychodynamic vs. cognitive-behavioral therapy, group vs. individual therapy), and the techniques employed in a specific session. A recent study has found that levels of synchrony, as well as the associations between synchrony and outcome may differ between types of treatment (Altmann et al., 2020)—specifically, in cognitive-behavioral therapy, as compared to psychodynamic therapy, movement synchrony was stronger and was more strongly associated with reductions in interpersonal problems, but less associated with the therapeutic alliance. There may well be similar differences in the extent to which different treatment modalities lead to different levels of inter-brain plasticity, or in the extent to which inter-brain plasticity is associated with outcome measures in these various modalities. Similarly, different treatment modalities might foster different types of synchrony (e.g., patient-led or therapist-led synchrony).

Some modes of treatment may foster less inter-brain synchrony, which should lead to less inter-brain plasticity. For example, in treatments which utilize virtual reality (Emmelkamp and Meyerbröker, 2021) or psychoactive drugs (De Gregorio et al., 2021) the therapist usually does not take part in the specific key activity (using a virtual reality device or a psychoactive drug) alongside the patient. This may result in less time spent in high inter-brain synchrony and reduce inter-brain plasticity, at least in the specific sessions in which these activities take place. Other techniques may increase generalization—for example, therapeutic techniques which attempt to simulate outside circumstances, such as imagery rescripting (Arntz, 2012) or role-playing (Kipper, 1986), may help inter-brain plasticity generalize to situations outside of the clinic and increase its impact. Biological factors may also come into play. For example, applying sleep deprivation as part of therapy (Dallaspezia and Benedetti, 2014) may reduce neural consolidation, and as a result reduce inter-brain plasticity. Of course, these ideas should first be examined by future research.

Finally, irrespective of their current ability to synchronize, some patients may have a reduced aptitude for inter-brain plasticity itself, as a result of certain psychological or neurological conditions. According to our model, these patients may gain little from psychotherapy. If such conditions exist, identifying them should be an important research focus.

### Implications in other contexts

Inter-brain plasticity as a mechanism of change has implications beyond traditional therapy sessions. First, complex plasticity dynamics may arise when more than two people are present, as in couples or group therapy. Research on group learning has established that groups of students are able to synchronize with a teacher and with one another (Bevilacqua et al., 2019), but synchrony was not associated with material retention. A recent study of physiological synchrony in couples therapy (Tourunen et al., 2020) highlights unique complexities that may arise in these situations; while in general physiological synchrony between couple members increased over the course of therapy, an increase which was associated with better outcomes, female clients’ outcomes improved when synchrony between male clients and female therapists *decreased*. These findings demonstrate that in a group setting, participants are not only in or out of synchrony with other participants, but might also be affected by relationships between other participants which do not involve them. Future studies could examine whether inter-brain synchrony in group therapy leads to inter-brain plasticity, look at the ways in which each participant’s ability to synchronize influences group processes, and examine the effects of observing other participants being in a high-synchrony interaction.

Second, inter-brain plasticity may have implications for therapist training. As mentioned above, a study by Zhang et al. (2020) has demonstrated that therapists who have completed their training had stronger synchrony with their patients than those just beginning, indicating that the ability to synchronize improves as one trains as a therapist. Future studies may find ways to fine-tune training programs to maximize this kind of improvement.

## Conclusion

The current review has presented evidence demonstrating that inter-brain plasticity may be an important mechanism of change in psychotherapy. Effective psychotherapy involves inter-brain synchrony, and repeated interpersonal interactions with high inter-brain synchrony can induce inter-brain plasticity, increasing the ability to synchronize in future interactions. This may be especially true for the core theory of mind network and the observation-execution system. Finally, inter-brain plasticity may underlie known outcomes of psychotherapy, such as improved coping with various psychological conditions which involve deficiencies in patients’ ability to synchronize as well as general improvements in patients’ interpersonal functioning. Thus, incorporating the inter-brain plasticity approach can offer new directions for the study of change in psychotherapy.

## Author contributions

HS and SS-T conceptualized the proposed model. HS wrote the first draft. SZ-M and SS-T offered critical revisions. All authors contributed to the article and approved the submitted version.

## Footnotes

1 While in the interest of brevity we sometimes discuss a general “ability to synchronize,” a person’s ability to synchronize is always context-dependent, as detailed here. We expand on this more below when discussing generalization.

2 The reason we used “inter-brain” with “synchrony” and “synchronization” but used “brain” with “coupling” is that “brain synchrony” usually refers to synchronization between two regions in the same brain. “Brain coupling” is almost exclusively used for inter-brain synchrony, and the search term “brain coupling” also captures papers with the term “inter-brain coupling”.
